# Supplementary material for: Tracheostomy care quality improvement in low- and middle-income countries: A scoping review
Source: PLOS Glob Public Health. 2023 Nov 9;3(11):e0002294. doi: 10.1371/journal.pgph.0002294 (PMC10635432; doi:10.1371/journal.pgph.0002294)
Supplement: S1 Appendix — (DOCX) [file pgph.0002294.s002.docx]

**S1 Appendix. Search Strategy**

**PUBMED**

QUERY

("Tracheostomy"[Mesh] OR tracheostom*[tw] OR "Tracheotomy"[Mesh] OR "tracheotom*"[tw])

AND

(Developing Countries[Mesh] OR "developing countr*"[tw] OR "under developed"[tw] OR "third world"[tw] OR "less developed"[tw] OR “Global Health”[Mesh] OR “low income”[tw] OR “lower middle income”[tw] OR "Africa"[Mesh] OR “africa”[tw] OR “afghanistan”[Mesh] OR “afghanistan”[tw] OR “burundi”[Mesh] OR “burundi”[tw] OR “burkina faso”[Mesh] OR “burkina faso”[tw] OR “central african republic”[Mesh] OR “central african republic”[tw] OR “congo”[Mesh] OR “congo”[tw] OR “eritrea”[Mesh] OR “eritrea”[tw] OR “ethiopia”[Mesh] OR “ethiopia”[tw] OR “guinea”[Mesh] OR “guinea”[tw] OR “gambia”[Mesh] OR “gambia”[tw] OR “liberia”[Mesh] OR “liberia”[tw] OR “madagascar”[Mesh] OR “madagascar”[tw] OR “mali”[Mesh] OR “mali”[tw] OR “mozambique”[Mesh] OR “mozambique”[tw] OR “malawi”[Mesh] OR “malawi”[tw] OR “niger”[Mesh] OR “niger”[tw] OR “rwanda”[Mesh] OR “rwanda”[tw] OR “sudan”[Mesh] OR “sudan”[tw] OR “sierra leone”[Mesh] OR “sierra leone”[tw] OR “somalia”[Mesh] OR “somalia”[tw] OR “syria”[Mesh] OR “syria*”[tw] OR “chad”[Mesh] OR “chad”[tw] OR “togo”[Mesh] OR “togo”[tw] OR “uganda”[Mesh] OR “uganda”[tw] OR “venezuela”[Mesh] OR “venezuela”[tw] OR “yemen”[Mesh] OR “yemen”[tw] OR “angola”[Mesh] OR “angola”[tw] OR “benin”[Mesh] OR “benin”[tw] OR “bangladesh”[Mesh] OR “bangladesh”[tw] OR “belize”[Mesh] OR “belize”[tw] OR “bolivia”[Mesh] OR “bolivia”[tw] OR “bhutan”[Mesh] OR “bhutan”[tw] OR “cote d’ivoire”[Mesh] OR “cote d’ivoire”[tw] OR “cameroon”[Mesh] OR “cameroon”[tw] OR “comoros”[Mesh] OR “comoros”[tw] OR “cabo verde”[Mesh] OR “cabo verde”[tw] OR “djibouti”[Mesh] OR “djibouti”[tw] OR “algeria”[Mesh] OR “algeria”[tw] OR “egypt”[Mesh] OR “egypt”[tw] OR “micronesia”[Mesh] OR “micronesia”[tw] OR “ghana”[Mesh] OR “ghana”[tw] OR “honduras”[Mesh] OR “honduras”[tw] OR “haiti”[Mesh] OR “haiti”[tw] OR “indonesia”[Mesh] OR “indonesia”[tw] OR “india”[Mesh] OR “india”[tw] OR “iran”[Mesh] OR “iran”[tw] OR “kenya”[Mesh] OR “kenya”[tw] OR “kyrgyz”[Mesh] OR “kyrgyz”[tw] OR “cambodia”[Mesh] OR “cambodia”[tw] OR “kiribati”[Mesh] OR “kiribati”[tw] OR “lao”[Mesh] OR “lao”[tw] OR “sri lanka”[Mesh] OR “sri lanka”[tw] OR “lesotho”[Mesh] OR “lesotho”[tw] OR “morocco”[Mesh] OR “morocco”[tw] OR “myanmar”[Mesh] OR “myanmar”[tw] OR “mongolia”[Mesh] OR “mongolia”[tw] OR “mauritania”[Mesh] OR “mauritania”[tw] OR “nigeria”[Mesh] OR “nigeria”[tw] OR “nicaragua”[Mesh] OR “nicaragua”[tw] OR “nepal”[Mesh] OR “nepal”[tw] OR “pakistan”[Mesh] OR “pakistan”[tw] OR “philippines”[Mesh] OR “philippines”[tw] OR “west bank”[Mesh] OR “west bank”[tw] OR “gaza”[Mesh] OR “gaza”[tw] OR “senegal”[Mesh] OR “senegal”[tw] OR “solomon islands”[Mesh] OR “solomon islands”[tw] OR “el salvador”[Mesh] OR “el salvador”[tw] OR “sao tome”[Mesh] OR “sao tome”[tw] OR “eswatini”[Mesh] OR “eswatini”[tw] OR “tajikistan”[Mesh] OR “tajikistan”[tw] OR “timor-leste”[Mesh] OR “timor-leste”[tw] OR “tunisia”[Mesh] OR “tunisia”[tw] OR “tanzania”[Mesh] OR “tanzania”[tw] OR “ukraine”[Mesh] OR “ukraine”[tw] OR “uzbekistan”[Mesh] OR “uzbekistan”[tw] OR “vietnam”[Mesh] OR “vietnam”[tw] OR “vanuatu”[Mesh] OR “vanuatu”[tw] OR “samoa”[Mesh] OR “samoa”[tw] OR “zambia”[Mesh] OR “zambia”[tw] OR “zimbabwe”[Mesh] OR “zimbabwe”[tw] OR “upper middle income”[tw] OR “albania”[Mesh] OR “albania”[tw] OR “american samoa”[Mesh] OR “american samoa”[tw] OR “argentina”[Mesh] OR “argentina”[tw] OR “armenia”[Mesh] OR “armenia”[tw] OR “azerbaijan”[Mesh] OR “azerbaijan”[tw] OR “belarus”[Mesh] OR “belarus”[tw] OR “belize”[Mesh] OR “belize”[tw] OR “bosnia and herzegovina”[Mesh] OR “bosnia and herzegovina”[tw] OR “botswana”[Mesh] OR “botswana”[tw] OR “brazil”[Mesh] OR “brazil”[tw] OR “bulgaria”[Mesh] OR “bulgaria”[tw] OR “china”[Mesh] OR “china”[tw] OR “colombia”[Mesh] OR “colombia”[tw] OR “costa rica”[Mesh] OR “costa rica”[tw] OR “cuba”[Mesh] OR “cuba”[tw] OR “dominica”[Mesh] OR “dominica”[tw] OR “dominican republic”[Mesh] OR “dominican republic”[tw] OR “equatorial guinea”[Mesh] OR “equatorial guinea”[tw] OR “ecuador”[Mesh] OR “ecuador”[tw] OR “fiji”[Mesh] OR “fiji”[tw] OR “gabon”[Mesh] OR “gabon”[tw] OR “georgia”[Mesh] OR “georgia”[tw] OR “grenada”[Mesh] OR “grenada”[tw] OR “guatemala”[Mesh] OR “guatemala”[tw] OR “guyana”[Mesh] OR “guyana”[tw] OR “iraq”[Mesh] OR “iraq”[tw] OR “jamaica”[Mesh] OR “jamaica”[tw] OR “jordan”[Mesh] OR “jordan”[tw] OR “kazakhstan”[Mesh] OR “kazakhstan”[tw] OR “kosovo”[Mesh] OR “kosovo”[tw] OR “libya”[Mesh] OR “libya”[tw] OR “malaysia”[Mesh] OR “malaysia”[tw] OR “maldives”[Mesh] OR “maldives”[tw] OR “marshall islands”[Mesh] OR “marshall islands”[tw] OR “mauritius”[Mesh] OR “mauritius”[tw] OR “mexico”[Mesh] OR “mexico”[tw] OR “moldova”[Mesh] OR “moldova”[tw] OR “montenegro”[Mesh] OR “montenegro”[tw] OR “namibia”[Mesh] OR “namibia”[tw] OR “north macedonia”[Mesh] OR “north macedonia”[tw] OR “palau”[Mesh] OR “palau”[tw] OR “paraguay”[Mesh] OR “paraguay”[tw] OR “peru”[Mesh] OR “peru”[tw] OR “russia”[Mesh] OR “russia”[tw] OR “russian federation”[Mesh] OR “russian federation”[tw] OR “serbia”[Mesh] OR “serbia”[tw] OR “south africa”[Mesh] OR “south africa”[tw] OR “st. lucia”[Mesh] OR “st. lucia”[tw] OR “st. vincent and the grenadines”[Mesh] OR “st. vincent and the grenadines”[tw] OR “suriname”[Mesh] OR “suriname”[tw] OR “thailand”[Mesh] OR “thailand”[tw] OR “tonga”[Mesh] OR “tonga”[tw] OR “turkey”[Mesh] OR “turkey”[tw] OR “turkmenistan”[Mesh] OR “turkmenistan”[tw] OR “tuvalu”[Mesh] OR “tuvalu”[tw])

FILTERS: year 2000 - 2022, English language, Full text

EXCLUDED: Abstracts

**EMBASE**

QUERY

'tracheostomy'/exp OR tracheostom*:ti,ab,kw OR 'tracheotomy'/exp OR tracheotom*:ti,ab,kw

AND

'low income country'/exp OR 'middle income country'/exp OR 'developing country'/exp OR ‘developing countr*’:ti,ab,kw OR ‘under developed’:ti,ab,kw OR ‘third world’:ti,ab,kw OR ‘less developed’:ti,ab,kw OR ‘Global Health’/exp OR ‘lower middle income’:ti,ab,kw OR 'africa'/exp OR ‘africa’:ti,ab,kw OR ‘afghanistan’/exp OR ‘afghanistan’:ti,ab,kw OR ‘burundi’/exp OR ‘burundi’:ti,ab,kw OR ‘burkina faso’/exp OR ‘burkina faso’:ti,ab,kw OR ‘central african republic’/exp OR ‘central african republic’:ti,ab,kw OR ‘congo’/exp OR ‘congo’:ti,ab,kw OR ‘eritrea’/exp OR ‘eritrea’:ti,ab,kw OR ‘ethiopia’/exp OR ‘ethiopia’:ti,ab,kw OR ‘guinea’/exp OR ‘guinea’:ti,ab,kw OR ‘gambia’/exp OR ‘gambia’:ti,ab,kw OR ‘liberia’/exp OR ‘liberia’:ti,ab,kw OR ‘madagascar’/exp OR ‘madagascar’:ti,ab,kw OR ‘mali’/exp OR ‘mali’:ti,ab,kw OR ‘mozambique’/exp OR ‘mozambique’:ti,ab,kw OR ‘malawi’/exp OR ‘malawi’:ti,ab,kw OR ‘niger’/exp OR ‘niger’:ti,ab,kw OR ‘rwanda’/exp OR ‘rwanda’:ti,ab,kw OR ‘sudan’/exp OR ‘sudan’:ti,ab,kw OR ‘sierra leone’/exp OR ‘sierra leone’:ti,ab,kw OR ‘somalia’/exp OR ‘somalia’:ti,ab,kw OR ‘syria’/exp OR ‘syria*’:ti,ab,kw OR ‘chad’/exp OR ‘chad’:ti,ab,kw OR ‘togo’/exp OR ‘togo’:ti,ab,kw OR ‘uganda’/exp OR ‘uganda’:ti,ab,kw OR ‘venezuela’/exp OR ‘venezuela’:ti,ab,kw OR ‘yemen’/exp OR ‘yemen’:ti,ab,kw OR ‘angola’/exp OR ‘angola’:ti,ab,kw OR ‘benin’/exp OR ‘benin’:ti,ab,kw OR ‘bangladesh’/exp OR ‘bangladesh’:ti,ab,kw OR ‘belize’/exp OR ‘belize’:ti,ab,kw OR ‘bolivia’/exp OR ‘bolivia’:ti,ab,kw OR ‘bhutan’/exp OR ‘bhutan’:ti,ab,kw OR ‘ivoire’/exp OR ‘ivoire’:ti,ab,kw OR ‘cameroon’/exp OR ‘cameroon’:ti,ab,kw OR ‘comoros’/exp OR ‘comoros’:ti,ab,kw OR ‘cabo verde’/exp OR ‘cabo verde’:ti,ab,kw OR ‘djibouti’/exp OR ‘djibouti’:ti,ab,kw OR ‘algeria’/exp OR ‘algeria’:ti,ab,kw OR ‘egypt’/exp OR ‘egypt’:ti,ab,kw OR ‘micronesia’/exp OR ‘micronesia’:ti,ab,kw OR ‘ghana’/exp OR ‘ghana’:ti,ab,kw OR ‘honduras’/exp OR ‘honduras’:ti,ab,kw OR ‘haiti’/exp OR ‘haiti’:ti,ab,kw OR ‘indonesia’/exp OR ‘indonesia’:ti,ab,kw OR ‘india’/exp OR ‘india’:ti,ab,kw OR ‘iran’/exp OR ‘iran’:ti,ab,kw OR ‘kenya’/exp OR ‘kenya’:ti,ab,kw OR ‘kyrgyz’/exp OR ‘kyrgyz’:ti,ab,kw OR ‘cambodia’/exp OR ‘cambodia’:ti,ab,kw OR ‘kiribati’/exp OR ‘kiribati’:ti,ab,kw OR ‘lao’/exp OR ‘lao’:ti,ab,kw OR ‘sri lanka’/exp OR ‘sri lanka’:ti,ab,kw OR ‘lesotho’/exp OR ‘lesotho’:ti,ab,kw OR ‘morocco’/exp OR ‘morocco’:ti,ab,kw OR ‘myanmar’/exp OR ‘myanmar’:ti,ab,kw OR ‘mongolia’/exp OR ‘mongolia’:ti,ab,kw OR ‘mauritania’/exp OR ‘mauritania’:ti,ab,kw OR ‘nigeria’/exp OR ‘nigeria’:ti,ab,kw OR ‘nicaragua’/exp OR ‘nicaragua’:ti,ab,kw OR ‘nepal’/exp OR ‘nepal’:ti,ab,kw OR ’pakistan’/exp OR ‘pakistan’:ti,ab,kw OR ‘philippines’/exp OR ‘philippines’:ti,ab,kw OR ‘west bank’/exp OR ‘west bank’:ti,ab,kw OR ‘gaza’/exp OR ‘gaza’:ti,ab,kw OR ‘senegal’/exp OR ‘senegal’:ti,ab,kw OR ‘solomon islands’/exp OR ‘solomon islands’:ti,ab,kw OR ‘el salvador’/exp OR ‘el salvador’:ti,ab,kw OR ‘sao tome’/exp OR ‘sao tome’:ti,ab,kw OR ‘eswatini’/exp OR ‘eswatini’:ti,ab,kw OR ‘tajikistan’/exp OR ‘tajikistan’:ti,ab,kw OR ‘timor-leste’/exp OR ‘timor-leste’:ti,ab,kw OR ‘tunisia’/exp OR ‘tunisia’:ti,ab,kw OR ‘tanzania’/exp OR ‘tanzania’:ti,ab,kw OR ‘ukraine’/exp OR ‘ukraine’:ti,ab,kw OR ‘uzbekistan’/exp OR ‘uzbekistan’:ti,ab,kw OR ‘vietnam’/exp OR ‘vietnam’:ti,ab,kw OR ‘vanuatu’/exp OR ‘vanuatu’:ti,ab,kw OR ‘samoa’/exp OR ‘samoa’:ti,ab,kw OR ‘zambia’/exp OR ‘zambia’:ti,ab,kw OR ‘zimbabwe’/exp OR ‘zimbabwe’:ti,ab,kw OR ‘upper middle income’:ti,ab,kw OR ‘afghanistan’/exp OR ‘afghanistan’:ti,ab,kw OR ‘albania’/exp OR ‘albania’:ti,ab,kw OR ‘american samoa’/exp OR ‘american samoa’:ti,ab,kw OR ‘argentina’/exp OR ‘argentina’:ti,ab,kw OR ‘armenia’/exp OR ‘armenia’:ti,ab,kw OR ‘azerbaijan’/exp OR ‘azerbaijan’:ti,ab,kw OR ‘belarus’/exp OR ‘belarus’:ti,ab,kw OR ‘belize’/exp OR ‘belize’:ti,ab,kw OR ‘bosnia and herzegovina’/exp OR ‘bosnia and herzegovina’:ti,ab,kw OR ‘botswana’/exp OR ‘botswana’:ti,ab,kw OR ‘brazil’/exp OR ‘brazil’:ti,ab,kw OR ‘bulgaria’/exp OR ‘bulgaria’:ti,ab,kw OR ‘china’/exp OR ‘china’:ti,ab,kw OR ‘colombia’/exp OR ‘colombia’:ti,ab,kw OR ‘costa rica’/exp OR ‘costa rica’:ti,ab,kw OR ‘cuba’/exp OR ‘cuba’:ti,ab,kw OR ‘dominica’/exp OR ‘dominica’:ti,ab,kw OR ‘dominican republic’/exp OR ‘dominican republic’:ti,ab,kw OR ‘equatorial guinea’/exp OR ‘equatorial guinea’:ti,ab,kw OR ‘ecuador’/exp OR ‘ecuador’:ti,ab,kw OR ‘fiji’/exp OR ‘fiji’:ti,ab,kw OR ‘gabon’/exp OR ‘gabon’:ti,ab,kw OR ‘georgia’/exp OR ‘georgia’:ti,ab,kw OR ‘grenada’/exp OR ‘grenada’:ti,ab,kw OR ‘guatemala’/exp OR ‘guatemala’:ti,ab,kw OR ‘guyana’/exp OR ‘guyana’:ti,ab,kw OR ‘iraq’/exp OR ‘iraq’:ti,ab,kw OR ‘jamaica’/exp OR ‘jamaica’:ti,ab,kw OR ‘jordan’/exp OR ‘jordan’:ti,ab,kw OR ‘kazakhstan’/exp OR ‘kazakhstan’:ti,ab,kw OR ‘kosovo’/exp OR ‘kosovo’:ti,ab,kw OR ‘libya’/exp OR ‘libya’:ti,ab,kw OR ‘malaysia’/exp OR ‘malaysia’:ti,ab,kw OR ‘maldives’/exp OR ‘maldives’:ti,ab,kw OR ‘marshall islands’/exp OR ‘marshall islands’:ti,ab,kw OR ‘mauritius’/exp OR ‘mauritius’:ti,ab,kw OR ‘mexico’/exp OR ‘mexico’:ti,ab,kw OR ‘moldova’/exp OR ‘moldova’:ti,ab,kw OR ‘montenegro’/exp OR ‘montenegro’:ti,ab,kw OR ‘namibia’/exp OR ‘namibia’:ti,ab,kw OR ‘north macedonia’/exp OR ‘north macedonia’:ti,ab,kw OR ‘palau’/exp OR ‘palau’:ti,ab,kw OR ‘paraguay’/exp OR ‘paraguay’:ti,ab,kw OR ‘peru’/exp OR ‘peru’:ti,ab,kw OR ‘russia’/exp OR ‘russia’:ti,ab,kw OR ‘russian federation’/exp OR ‘russian federation’:ti,ab,kw OR ‘serbia’/exp OR ‘serbia’:ti,ab,kw OR ‘south africa’/exp OR ‘south africa’:ti,ab,kw OR ‘st. lucia’/exp OR ‘st. lucia’:ti,ab,kw OR ‘st. vincent and the grenadines’/exp OR ‘st. vincent and the grenadines’:ti,ab,kw OR ‘suriname’/exp OR ‘suriname’:ti,ab,kw OR ‘thailand’/exp OR ‘thailand’:ti,ab,kw OR ‘tonga’/exp OR ‘tonga’:ti,ab,kw OR ‘turkey’/exp OR ‘turkey’:ti,ab,kw OR ‘turkmenistan’/exp OR ‘turkmenistan’:ti,ab,kw OR ‘tuvalu’/exp OR ‘tuvalu’:ti,ab,kw

FILTERS: years 2000-2022, English language, Articles and Reviews

EXCLUDED: Conference Abstract, Letter, Conference Review, Article in Press, Conference Paper, Editorial, Note, Short Survey, Erratum, Tombstone
