# Supplementary material for: Tracheostomy care quality improvement in low- and middle-income countries: A scoping review
Source: PLOS Glob Public Health. 2023 Nov 9;3(11):e0002294. doi: 10.1371/journal.pgph.0002294 (PMC10635432; doi:10.1371/journal.pgph.0002294)
Supplement: Attachment — Submitted filename: 20230917 Response to Reviewers.docx [file pgph.0002294.s003.docx]

**Response to Reviewers**

**Reviewer #1**

1. **The article is very relevant and needed. The authors have reviewed the existing literature very well.
   I would like to advise one thing, the article is very lengthy and at some points this makes it less interesting for the readers, making the article little short and to the point will help improve quality.**

We thank the reviewer for this suggestion. We have worked to make the paper more concise, especially focusing on the results section. The manuscript (not including the abstract) has been cut down from 3314 words to 2979 words.

**Reviewer #2**

1. **General**

**This paper addresses an important gap in available literature regrading tracheostomy care in LMICs. A scoping review seems appropriate. Exclusion of non-English publications is a significant limitation given the areas of the world the authors are trying to address.**

We thank the reviewer for this suggestion. We agree that this is a limitation, which is included in the discussions section.

To further assess how many non-English papers the search may have missed, we re-ran the search with other languages. Using the same PubMed search terms, we reperformed the search strategy (2000-2022, inclusion of full text, exclusion of abstracts) and this time included the official United Nations languages other than English (Arabic, Chinese, French, Russian, and Spanish). There were 34 results with the following PubMed ID numbers:

37554082

36571521

36542581

36413169

36058661

35982007

35927044

35620174

34886628

33047711

32074748

30481918

30440148

30060347

29953099

29742220

29333814

28914829

28838625

26711073

26608271

26161221

25637894

25572889

23827268

23583718

22093637

21029685

16886543

16620596

16483491

12760795

10668080

1286582

The only relevant publication following title and abstract screening was the following discussing developing consensus guidelines.

Similar to the two papers included in the scoping review, the abstract of this paper which was available in English discussed a multidisciplinary team approach and did not report clinical outcomes. These were similar findings to the included publications on tracheostomy consensus guidelines in LMICs.

Given this, we suspect that the search strategy in English has captured the majority of the papers needed to synthesize the gaps in the literature regarding tracheostomy care in LMICs. We still do agree that this is a limitation, which is noted in the manuscript. Given the limited literature available and the search of PubMed to include languages outside of English, it is unclear whether additional searches will change the main findings and conclusions. Per the editor’s discretion, we are happy to redo the search if this is recommended.

We hope with increased research in the area, future systematic reviews will expand to include papers beyond the English literature.

2. **Introduction/ Background
   The authors appear to extrapolate rates of procedure and complications from HICs to LMICs, as there are no citations to support their claims of complication rates in LMICs. Having some support for the incidence of tracheostomy and associated complications would help make this stronger. Are rates of tracheostomy in LMICs as high as in HICs? This is assumed based on the overall higher global population. If the authors are making this assumption, it should be explicit.**

We thank the reviewer for this suggestion on two areas—estimates of the tracheotomy volume and the complication rates from LMICs.

For the volume of tracheotomies performed in LMICs, we do agree that procedural volume would be an estimate and extrapolation from HICs. To clarify, this, we have changed the following statement in the background, lines 81-82 on the tracked changes version and 61-62 on the cleaned version.

Prior version:

*An estimated 250,000 procedures are performed annually in high income countries (HICs), and numbers are expected to increase in both HICs and low and middle-income countries (LMICs).(2–5)*

Updated version:

*An estimated 250,000 procedures are performed annually in high income countries (HICs) (2–5).*

The complications rates cited are from two studies from HICs and one from an LMIC (Uganda). This has been clarified in the text of the background section, lines 84-86 on the tracked changes version and 64-66 on the cleaned version.

Prior version:

*Published literature reports complication rates ranging from 4% to 66% and all-cause mortality rates up to 22%.(4,6,7)*

Updated version:

*Published literature from both HIC and low and middle-income country (LMIC) settings reports complication rates ranging from 4% to 66% and all-cause mortality rates up to 22%(4,6,7).*

3. **Results/Discussion
   The logic model is a helpful figure and contribution to further studies. While the goal of the logic model is different than the Global Tracheostomy Collaborative drivers of quality improvement (as outlined by the authors), it would bring the paper together to frame the logic model within the goals of the GTC, or at least tie them together somehow. While the goals are admirable, it seems that more wheels are being invented which ultimately undermines efforts to achieve these things.**

We thank the author for this suggestion. We agree that a logic model incorporating the GTC principles more explicitly would enhance the cohesiveness of the paper. We have therefore modified figure 2 to incorporate the GTC’s five key drivers of QI as seen in the updated figure below and in the text in the discussion section, lines 813-815 on the tracked changes version and 299-301 on the cleaned version.

Prior version and figure:

*Given the complex challenges associated with tracheostomy care in LMICs, an implementation framework is one approach to address the comprehensive nature of building a successful tracheostomy care program (Figure 2).*

Updated version and figure. Changes to the figure outlined in blue:

*Given the complex challenges associated with tracheostomy care in LMICs, an implementation framework is one approach to address the comprehensive nature of building a successful tracheostomy care program that can incorporate the GTC key drivers of QI in the implementation strategies (Figure 2).*
